# Knowledge, attitudes, and referral practices for smokers to a state tobacco quitline in a federally qualified healthcare center: Healthcare provider perspectives

**DOI:** 10.18332/tpc/191728

**Published:** 2024-10-30

**Authors:** Alicia K. Matthews, Cherdsak Duangchan, Jennifer Afuko, Hope Opuada, Geri Donenberg

**Affiliations:** 1 School of Nursing, Columbia University, New York, United States; 2 College of Nursing, University of Illinois Chicago, Chicago, United States; 3 Center for Dissemination and Implementation Science, College of Medicine, University of Illinois Chicago, Chicago, United States

**Keywords:** state tobacco quitlines, healthcare providers, federally qualified health centers, treatment referral

## Abstract

**INTRODUCTION:**

Federally qualified healthcare centers (FQHC) treat a large population of low-income patients disproportionately burdened by tobacco use. This study investigated healthcare providers’ knowledge, attitudes, and referral patterns of patients who smoke to a state tobacco quitline.

**METHODS:**

The study used a descriptive-qualitative design. In-depth interviews were conducted in 2021 with a sample of healthcare providers recruited from a federally qualified healthcare center (FQHC) in a large city in the Midwest. The interviews were guided by a standardized moderator's guide and lasted 30–45 minutes. Written informed consent was obtained before each interview, and participants completed a brief self-administered survey.

**RESULTS:**

Among the 25 participants, 92% were female and 44% were Black. Participants included medical providers (52%), behavioral health providers (16%), and other types of providers (32%). Participants’ age and work experience averaged 41.5 and 5.25 years, respectively. Only 32% of providers reported having specialty training in smoking cessation or addiction counseling. Over half (52%) of the participants never or rarely referred patients to the Illinois Tobacco Quitline (ITQL). Providers reported several barriers to referring patients to the ITQL, including limited knowledge about services offered, time constraints, difficulties with the referral process, and lack of feedback between providers and the ITQL. Further, providers described patient-related barriers, including low motivation to quit smoking, language barriers, and failure of patients to respond to calls from the quitline. Recommendations were described for improving patient and provider education, referral processes, and increasing bi-directional communication between providers and the quitline.

**CONCLUSIONS:**

Providers identified numerous barriers to referring patients for smoking cessation treatment. Addressing the identified barriers requires a multi-faceted approach involving education, streamlined processes, supportive infrastructure, and patient-centered interventions to strengthen provider use and satisfaction with the available resources.

## INTRODUCTION

Over the past fifty years, tobacco prevention and control activities have significantly reduced the number of new and current smokers^1^. Estimates suggest that approximately 12.5% of adults in the US are current smokers, down from about 25% in 1993^2^. Despite these gains, smoking rates remain elevated among individuals from lower socioeconomic backgrounds. For example, in the US, those earning <$35000 a year have a smoking prevalence rate of 20.2%^3^. Also, people living in poverty smoke for nearly twice as many years as those with incomes above the poverty line^3^. Common factors contributing to continued tobacco use in this population are related to social determinants of health, including a lower level of education, fewer social and community norms that deter smoking, and a higher stress level^4^. Low-income individuals are also less likely to have insurance coverage, a regular source of care, and access to preventative health services such as tobacco cessation treatments. Many low-income patients rely on safety-net hospitals and clinics within their communities, which may be under-resourced to address their needs^4^. Indeed, as of 2018, only 15 state Medicaid programs entirely covered tobacco cessation services for enrollees in traditional Medicaid^5^. Hence, addressing the tobacco cessation needs of low-income smokers remains a significant public health priority nationally and internationally.

### Tobacco quitlines

The World Health Organization (WHO) Framework Convention on Tobacco Control (FCTC) has identified the availability of free tobacco quitlines as one of several strategies for reducing global morbidity and mortality from tobacco use. In the US, federal programs have sought to address barriers to receiving smoking cessation services among low-income and other population groups by providing access to free or low-cost smoking cessation services. In 2004, a single nationwide 1-800 portal (1-800-QUIT-NOW) was developed to give smokers uniform access to state quitlines^6^. In all 50 states, quitlines are telephone-based tobacco cessation services that help tobacco users quit or decrease their smoking by providing counseling, nicotine replacement, referrals to local cessation programs, and self-help materials sent via mail or online^7,8^. Approximately 500000 smokers contact a state tobacco quitline yearly, with over 126000 callers quitting because of treatment^6^.

In 2008, the Public Health Service (PHS) Clinical Practice Guideline, Treating Tobacco Use and Dependence, listed state tobacco quitlines as effective and recommended^9^. The potential impact of quitlines comes from their capacity to deliver evidence-based treatment for smokers at scale cost-effectively^6^. Further, quitline services are free to most smokers and are offered over the phone, which reduces substantial barriers to treatment for low-income smokers^6^. Despite the benefits of state quitlines, utilization rates are modest, especially among low-income individuals. According to the North American Quitline Consortium’s annual survey of quitlines, just under 1% of adults who smoke in the US used a quitline in 2019^10^.

Health providers serving low-income patients are essential in linking high-risk smokers to effective treatments such as state tobacco quitlines. For example, Federally Qualified Health Centers (FQHCs) provide comprehensive health services to economically disadvantaged populations in rural and urban communities across the US^11^. In the US, more than 28 million – one in three low-income persons who live below 200% of the federal poverty level – receive medical care at an FQHC^12^. Studies show an estimated median prevalence rate of 29.3% tobacco use among FQHC patient populations^12^.

Several barriers exist to consistently delivering smoking cessation treatments, including referrals to state quitlines by providers in high-burden clinics. For example, Gonzales et al.13 identified barriers to providing smokers in FQHC clinics with tobacco cessation assessment and interventions, including the Nebraska Tobacco Quitline. These authors found that providers and non-provider clinical staff reported a lack of awareness, time, competing demands, electronic health records (EHR) that do not facilitate recommendations/referrals, insufficient training, and limited staff as primary barriers to treatment at the FQHC setting^13^.

Research examining interventions to improve patient linkage to state tobacco quitlines is emerging. For example, the Reach, Effectiveness, Adoption, Implementation, and Maintenance framework was used in a study to assess the implementation of a healthcare system change carried out across 30 primary care clinics within the University of Wisconsin Health system to address unmet needs of smokers in obtaining tobacco cessation treatments, including a linkage to the Wisconsin Tobacco Quitline (WTQL)^14^. The healthcare system employed standard, computer-based training to implement EHR and clinic workflow changes to facilitate electronic referrals, whereas previously, they faxed patient referrals to the WTQL. The EHR data captured assessment rates of patients’ readiness to quit and quitline referral 4 months before and 8 months after implementation. Results showed an increase in assessment in readiness to quit (24.8% to 93.2%) and an increase in quitline referrals (1.7% to 11.3%), with 3.6% of those patients connecting with the WTQL after implementation^14^.

This study sought to understand providers’ attitudes and behaviors concerning quitline services and referrals in a large urban FQHC. Specifically, we assessed providers’ knowledge about the Illinois state tobacco quitline (ITQL) services in the FQHC context, examined typical referral patterns to the ITQL among FQHC providers, and identified and explored barriers providers encounter when referring patients to the ITQL. The findings can inform targeted implementation strategies to improve quitline utilization and support smoking cessation efforts in this vulnerable population.

## METHODS

### Study design

The study used a descriptive-qualitative design^15,16^. Recruitment and data collection took place between August and October 2021. The Institutional Review Board of the University of Illinois Chicago (IRB # 2021-0578) approved the study.

### Setting

The study was conducted at Mile Square Health Center (MSHC), a federally qualified health center (FQHC) affiliated with the University of Illinois Hospital in Chicago, Illinois. MSHC consists of six primary care clinics in high-poverty neighborhoods with documented health inequalities associated with chronic disease. Annually, the clinic serves more than 40000 patients, most living at or below the federal poverty level (98%)^17^.

### Recruitment and enrollment

A volunteer convenience sample of providers was recruited from all six MSHCs using posted flyers and outreach via the clinic *listserv*. Eligibility requirements were: 1) aged ≥21 years, 2) a healthcare provider at MSHC, 3) English-speaking, and 4) able to provide informed consent. Interested individuals contacted the study team directly, and eligible individuals were scheduled for an interview. Recruitment and enrollment occurred continuously until the total sample size was achieved.

### Data collection procedures

All participants (n=25) completed the informed consent document and a brief (5–10 minute) demographic survey in REDCap before their interview. Semi-structured in-depth interviews were conducted in person (n=15), over the phone (n=9), or via Zoom (n=1). A standardized moderator’s guide was developed based on primary objectives and study research questions. Sample questions/items included: ‘How often do you refer your patients who smoke to the state quitline?’, ‘Please describe the procedures used by providers in your practice to refer patients to the quitline.’, and ‘How does the typical patient respond to your recommendation for a quitline referral?’. The interviews took 30–45 minutes (mean=40 minutes). Consistent with recommended approaches^18^, trained interviewers conducted and audio-recorded each interview and participated in a post-session debriefing to highlight significant themes. Next, audio recordings were transcribed verbatim by an independent transcriber and reviewed by two team members for accuracy. Audio recordings were destroyed after transcription was completed and verified. Participants received a $50 gift card for completing the study.

### Data analysis

Data analysis for this study used descriptive statistics to summarize the responses to the demographic questionnaire. Frequencies, means, and standard deviations were computed using SPSS, version 19 (IBM Corporation, Armonk, NY). We used deductive thematic analysis to analyze the interviews^18^. Codes were created based on the moderator’s guide and the Consolidated Framework for Implementation Research (CFIR)^19^. The CFIR is a widely recognized framework that provides a comprehensive approach to understanding barriers and facilitators to implementation processes. It encompasses five domains: intervention characteristics, outer setting, inner setting, characteristics of individuals, and the process of implementation. To ensure the validity and reliability of qualitative data, we used the Lincoln and Guba^20^ four criteria: credibility, dependability, confirmability, and transferability. The authors conducted an iterative process, individually reviewing the codes, categories, and themes. Subsequently, the authors met to discuss and document analytic insights, assumptions, and decisions related to the data analysis process. Thematic saturation (i.e. when no additional themes are generated in subsequent rounds of data collection)^21^ was reached with our sample.

## RESULTS

### Participant characteristics

Participants’ demographic characteristics are summarized in Table 1. Among the 25 participants, 92% were female, 44% were Black, 52% were medical providers, and 60% worked at the largest of the FQHC’s six primary care locations. Participants’ age and work experience averaged 41.5 (SD=9.4) and 5.25 (SD=5.9) years, respectively. Over two-thirds of participants (68%) had not received specialty training in smoking cessation or other addiction counseling. Furthermore, more than half (52%) reported never or rarely referring patients who smoked to the state tobacco quitline.

**Table 1:** Characteristics of providers from a federally qualified healthcare center who participated in a descriptive qualitative design study conducted in 2021 (N=25)

*Characteristics*	*n (%)*
**Age** (years), mean (SD), range	41.5 (9.4), 25–62
**Work experience** (years), mean (SD), range	5.25 (5.92), 1–22
**Gender**	
Female	23 (92.0)
Male	2 (8.0)
Race/ethnicity*	
Black	11 (44.0)
White	8 (32.0)
Hispanic/Latino	3 (12.0)
Other	4 (16.0)
Prefer not to answer	2 (8.0)
**Role**	
Medical provider (physician, physician assistant, nurse practitioner)	13 (52.0)
Behavioral health provider	4 (16.0)
Other (pharmacist, licensed practical nurse, certified medical assistant, medical assistant, recovery specialist)	8 (32.0)
**Setting**	
Main site	15 (60.0)
Back of the Yards	3 (12.0)
Cicero	4 (16.0)
Humboldt Park	2 (8.0)
Prefer not to answer	1 (4.0)
**Specialty training in smoking cessation or other addiction counseling**	
No	17 (68.0)
Yes	8 (32.0)
**Refers patients who smoke to the Illinois Tobacco Quitline**	
Never	4 (16.0)
Rarely	9 (36.0)
Sometimes	4 (16.0)
Often	4 (16.0)
Very often	3 (12.0)
Prefer not to answer	1 (4.0)

* Results >100% due to multiple choice.

### Qualitative results

The section below summarizes the qualitative findings related to provider attitudes, referral patterns, barriers to patient referral, patient-level factors influencing referral, and recommendations for improving the use of the ITQL among providers. Below, we describe each primary theme and subthemes, with illustrative quotations from the interviewees (coded for anonymity as I#), as appropriate.

### Characteristics of individuals: Providers

*Provider knowledge and attitudes*

Providers generally expressed positive attitudes about the ITQL as a smoking cessation resource for patients receiving care at MSHC. They reported receiving positive information about the ITQL from other clinicians and patients who have used the service. When providers were asked to identify the perceived benefits of the ITQL, they noted the expertise of quitline counselors, the availability of free counseling and nicotine replacement therapies, and the availability and flexibility of the services offered. Further, providers noted the value of having quitline information to share with patients who were not yet ready to quit smoking but might benefit from it:

*‘I have heard the clinicians talk about it fondly because they [Quitline counselors] shoulder the expertise for treatment. I’ve also heard patients get free products like nicotine patches. That appeals to a lot of patients.’* (I13)

*‘So, my patients that have used it have been happy. I must be honest. I can’t get everybody to use it. But, the patients that have used it appreciate the flexibility.’* (I3)

*‘It’s a great free resource that they could use at any time. It would be a resource that they could hang onto for whenever they felt ready to quit.’* (I6)

*Patterns of patient referral to ITQL*

Despite positive attitudes about the quitline, most providers reported infrequently referring patients to the ITQL. Those who referred patients reported their most common practice was to advise the patient to quit smoking and give them ITQL-related materials (such as business cards, flyers, or handouts). However, patients were responsible for calling the quitline directly after their clinic appointment:

*‘Well, I give them the business card and let them know to call, that they can give them more information about quitting tobacco or other options.’* (I7)

*‘I would just write the quitline number on their after-visit summary, or sometimes there were times where we have education on smoking cessation, and the quitline number would be in there, so I would just highlight it so they would have it.’* (I2)

‘*I have the business card, the Quitline business card. I have a stack of them that sit at my desk, and that’s what I give the patient.’* (I3)

In addition to providing patients with the ITQL information and materials, a few providers completed the required paperwork for direct referral and faxed it to the ITQL:

*‘We have been partnering with Illinois Tobacco Quitline. We received an in-service and materials from them. As I said, I give them the literature, fill out the form, and fax it to the Quitline.’* (I4)

*‘So, I would refer using the referral form. I would I fax it out, and then I give it to my clerk so they can upload it into the patient’s medical records.’* (I10)

### Characteristics of individuals: Patients

*Low-patient motivation to quit*

Providers identified patient characteristics impeding patients’ ITQL use. The first was low motivation among patients to engage in smoking cessation:

*‘So, they’ll take the card and tell me they’ll call. But when I see them again, and I’ll ask them, “Are you still smoking?” they say, “Yeah, I’m still working on it. But it’s hard”.’ So, that’s the response I get.’* (I18)

*Difficulties reaching patients*

Providers noted the failure of the quitline counselor to reach the patient after a referral. Providers stated that patients often did not recognize the ITQL counselor’s telephone number, missed their telephone calls, or did not check their voicemail:

*‘They had a smoking cessation navigator, and this person would refer them to the quitline. But some of the times patients wouldn’t answer [the quitline call] because they didn’t recognize the number.’* (I11)

*‘I was checking with some patients to see if they had gotten a call, and patients would sometimes say no. I don’t know if that’s because the Quitline never called them or if they just missed the call and didn’t listen to a voicemail.’* (I4)

*Language and literacy levels*

Patient language skills were listed as barriers to patients’ ITQL use. FQHC settings serve patients with low SES and diverse backgrounds, so some patients need help reading and understanding English. One participant stated that having Spanish-language resources and staff would ease this barrier for patients:

*‘We need to stop assuming people know how to read. Nine times out of 10, people just don’t read. They just don’t.’* (I5)

*‘It would help to have brochures in English and Spanish. They should have another line they can call and ask for somebody that speaks Spanish.’* (I8)

### Inner setting

*Provider knowledge gaps*

When asked why they did not refer patients to the ITQL, providers indicated a lack of knowledge about the quitline, namely when and how to refer patients, what services were available, what patients could expect from the ITQL (its processes or procedures), or how effective the ITQL would be. Also, some providers preferred referring patients to smoking cessation services in their clinic rather than to the ITQL:

*‘I don’t know how that would work, how the practitioner would get the information to the Quitline.’* (I3)

*‘I really don’t know how effective they are.’* (I8)

*‘No, I have not. I just not having a lot of knowledge about the services and what they can provide.’* (I9)

*‘I’m not certain who would need to be referred to that line if they’re a Mile Square patient because they can get assistance during their regular care.’* (I24)

*Provider time constraints*

Time constraints were cited as barriers to educating and referring patients to the ITQL. These constraints often resulted from heavy workloads, patients’ complex needs, and an overall clinic workflow that diverted their attention away from activities related to smoking cessation:

*‘I just know that it’s a resource that I could use. And if I’m being honest, I have not done my due diligence to look into it. And it’s not because I don’t want to or that I’m not interested. I’m stretched so thin already just taking that extra time to do so sometimes becomes difficult.’* (I5)

*‘Usually because of the flow. We just ask them questions, and by the time we’re done with our questionnaires and our information, the doctor is ready for them.’* (I19)

### Outer setting

*Referral processes*

Providers described the referral procedure to the ITQL as cumbersome, especially the complex paperwork and process. One participant reported insufficient system linkage between the clinical setting and the ITQL. Several participants expressed the need for a more efficient process:

*‘I understand that there is now a process in place to streamline Quitline referrals. I welcome that because filling out the form and faxing it in the midst of everything else is a little cumbersome.’* (I4)

*‘It was easier when it was built into the patient education forms that are already in the EMR.’* (I20)

*‘The form itself is annoying. And they told us they were going to upgrade it. We have a community health worker. So, I should probably have our community health worker do it [refer patients].’* (I4)

*Communication and follow-up*

Providers also identified the need for more communication between the ITQL and MSHC. Provider-initiated follow-up is optional at MSHC, and no formal data-sharing system has been established between the two systems. As a result, providers expressed a desire for better communication about whether patients accessed quitline services and patient outcomes such as quit status:

*‘I have actually never, ever heard from the Quitline.’* (I1)

*‘Sometimes, they use the Quitline, and we never hear about it again. Follow-ups aren’t required unless the patient wants them. So, if they connect with the Quitline and that’s been helpful to them, I might not hear from them again.’* (I21)

*‘I hope that they did, but I’m not sure. Once they leave, I don’t know if they do call or not.’* (I7)

One participant shared a positive experience contacting the ITQL to follow up on a patient referral:

*‘I called the quitline once. I wanted to find out if the people we’re referring over were following up. She did give me some follow up, and they were able to run reports.’* (I10)

### Process of implementation: Provider recommendations

*Integration of referral processes into usual care*

Providers offered recommendations for promoting ITQL linkage for patients. For example, they suggested integrating referrals to the quitline during the clinical appointment or having someone on site who can provide cessation services. Integrating quitline referral processes into usual care would allow providers and quitline staff to establish relationships and trust with patients, increasing the likelihood that patients engage with quitline services:

*‘It’s easier when we can connect the patient right in that moment. Once they leave [the clinic] and they’ve got a number to call or website to look at, it’s a lower likelihood of it happening.’* (I11)

*‘I think it’s best for patients to receive smoking cessation information where you receive your other services. Having a person that they can see within the clinic makes it more personal. Then later, after you’ve established a relationship, calling the person on the phone is okay.’* (I2)

*Bi-directional information exchange*

Providers suggested establishing efficient communication and information exchange systems between the ITQL and MSHC. Improved communication would permit providers to stay informed about their patients’ smoking status. Providers felt that access to this information would help them facilitate patients’ connection to and ongoing engagement with the quitline:

*‘I know they don’t have access to make a note into the patient’s chart. But if there’s some standardized thing, they can fax the provider. That way, we can review it and upload it to the patient’s chart. It would help to have this collaboration of care so we know what’s going on when we do the follow-up with the patient.’* (I9)

*Better understanding of patient preferences*

Finally, providers recommended considering patient preference when referring them to the ITQL. Some patients preferred to be called directly from the ITQL. However, others prefer to initiate the phone call themselves and perceive the ITQL phone calls as intrusive. Given their differing experiences with patients, the providers disagreed on whether the best approach was for patients to call or for the quitline to contact them:

*‘Sometimes patients are just so happy that people reach out to them. However, some patients feel like a call from the Quitline is intrusive. I respect it, but just getting the phone call could be helpful.’* (I3)

*‘Sometimes patients prefer to be called instead of trying to find the information. As soon as they leave the clinic, they’ll forget about everything. But, if someone calls them and reminds them, they’re like, “Oh, okay”.’* (I19)

## DISCUSSION

Provision of smoking cessation services is mandated for all FQHCs^13^, yet consistent delivery of such treatments remains a challenge. Brief evidence-based smoking cessation treatments exist, such as Ask, Advise, and Refer^22^, but providers often lack the knowledge, confidence, and time to deliver them^13^. To address this issue, efforts have been made to enhance partnerships between national stop-smoking resources and individual healthcare provider systems to facilitate referrals by healthcare providers to these national resources. This study examined FQHC providers’ knowledge, attitudes, and referral practices related to the ITQL, a state tobacco quitline. Results underscored individual provider and patient-level characteristics, inner and outer setting factors, and processes related to implementation as barriers to consistent referrals for smoking cessation services.

Consistent with the CFIR framework^19^, findings revealed individual provider characteristics as barriers to referring patients to the ITQL. Providers recognized the benefits of smoking cessation treatments but lacked knowledge about quitline services. Likewise, despite awareness of high smoking rates among their patients, referral rates to the ITQL were low. Even when providers began the referral process during the clinic appointment (e.g. provided business cards), most patients were expected to contact the quitline themselves. Interestingly, MSHC has an established relationship with the ITQL. However, the providers reported insufficient knowledge about quitline’s services, dissatisfaction with the referral process, and no feedback about their patients’ quitline engagement. These factors emerged as barriers to consistent referrals to the ITQL.

Individual patient-level characteristics were also reported as barriers, including low motivation to quit smoking. Overcoming patient-level barriers can be addressed through patient education, including developing educational materials emphasizing the benefits of quitting smoking and highlighting the services provided by tobacco quitlines. These educational materials can be distributed in waiting areas or during patient visits. Training healthcare providers in motivational interviewing may also address low patient motivation^23^. Indeed, motivational interviewing has been used extensively to engage patients in discussions about smoking cessation and could be used to motivate them to utilize tobacco quitline services^23^. Finally, it is essential to recognize and address cultural and language barriers impacting patients’ willingness to engage with tobacco quitlines. Several prior studies have identified the benefits of tailoring smoking resources to the needs of high-risk groups^24^ and the importance of language accessibility to increase patient engagement in health promotion behaviors^25^.

Aspects of inner and outer settings emerged as barriers to referring patients to the ITQL. Study findings underscore the importance of addressing provider knowledge gaps to improve referral processes for smoking cessation services within FQHCs. State tobacco quitlines can play an essential role in educating providers by reinforcing the effectiveness of brief counseling (Ask, Advise, and Refer)^22^ and information about the services offered by the quitline. Continuing medical and educational credits can be obtained from in-services offered by state quitlines or taped webinars that can be accessed on demand. As noted by providers interviewed for this study, state quitlines also have freely available patient and provider educational materials that can be downloaded from websites. The need to streamline referral processes and create systems to facilitate bi-directional communication was noted as an outer setting factor impacting the linkage of patients to the ITQL. By addressing these barriers, FQHCs may strengthen their capacity to support patient linkages to evidence-based interventions.

### Strategies for addressing barriers to quitline referral

Strategies to strengthen patients’ quitline use will require attention to multi-level targets. Four are suggested below: provider education, streamlining referral processes, information exchange and data sharing between providers and quitlines, and leveraging the patient portal to provide patient education and monitor referral uptake.

Comprehensive provider education about the services and advantages of quitlines is a crucial first step to increasing patient referrals. Our study findings suggest that healthcare providers would benefit from greater awareness of available resources and training on referring patients to quitlines. Providers often have limited time during patient visits, impeding discussions of smoking cessation options and referrals to tobacco quitlines. However, state tobacco quitlines could collaborate with FQHCs to create concise, evidence-based materials for providers to facilitate patient linkage and referral. These educational materials should highlight the benefits of tobacco quitlines and explain the referral process in a simple and easily understandable format.

A second strategy should establish more streamlined referral processes. Current practices are overly complex and time-consuming. Simplifying and streamlining the process can overcome these barriers, particularly by incorporating tobacco quitline referral options within EHR systems. Referral materials should describe standardized guidelines and steps to ensure efficient referral procedures and specify eligibility criteria, contact information, and necessary documentation. Alternatively, dedicated support staff, such as tobacco cessation coordinators or nurses, can work directly with patients to facilitate the referral process, assist with paperwork, provide patient information, and follow up on referrals. Dedicated staff would reduce the administrative burden on providers and enable referrals with minimal effort and time.

Thirdly, study participants recommended strengthening communication between FQHC providers and quitline counselors using a formal feedback mechanism, information exchange system, and data sharing. More effective communication would alert providers to their patients’ smoking status and progress toward cessation, thereby improving providers’ ability to encourage patient persistence in cessation efforts and engagement with the quitline. One method to improve information exchange and communication across settings is to use the EHR and eReferrals. EHR-supported eReferrals will help maintain smooth clinic workflows in healthcare settings and minimize providers’ cognitive burden^26^. For example, in a study using data from the EHR at the University of California, Davis Health Systems to send a tobacco eReferral to state quitline^26^, results showed that over 3 years, 16083 encounters with smokers resulted in 1137 (7.1%) eReferral orders. Treatment reach was 1.6% for quitline services, and referrals doubled after inpatient eReferral order sets were implemented. Among first-time eReferral patients, 12.2% had a 6- to 12-month follow-up visit at which they were documented as non-smoking^26^.

Finally, time constraints are repeatedly reported as barriers to providers addressing patient’s smoking cessation needs. FQHCs could leverage patients’ health portals to address issues related to time constraints. For example, providers should continue to advise patients to quit smoking and to refer patients to the ITQL. Also, with the referral to the ITQL, an automated message could be sent to the patients’ health portal that serves as a reminder that their provider has referred them to the quitline, provides standardized information about the quitline’s procedures and services, and lists the quitline’s telephone number so patients can recognize it when the quitline calls or they can phone the ITQL directly, based on preferences. Further, the electronic health record could be programmed to send the referral to the state tobacco quitlines and send automated messages to the patient’s portal as a reminder. The sent referral could also trigger a message to patients within a specified timeframe (e.g. two weeks after the referral) to determine whether a connection was made and if barriers were encountered. A community health worker or smoking cessation counselor could monitor patient messages and provide information and resources as needed.

### Limitations

It is essential to acknowledge the following limitations. Firstly, data are limited to a single healthcare system in a large urban area with providers from multiple clinic locations within the FQHC. Data from one healthcare system may limit the generalizability of the findings to FQHCs in different geographical locations, particularly those in rural or suburban areas. Providers in these areas may face distinct barriers and require different strategies to facilitate patient referrals to quitline services. Replication of the study in diverse FQHC settings is necessary to identify additional barriers or facilitators.

This study examined provider perspectives only – other factors, such as patient-reported preferences and organizational policies, likely influence quitline referrals. Future research could explore additional factors and patient perspectives to better understand the complex influences on quitline referrals. Research is also needed to expand the understanding of quitline referral practices and optimize their integration into FQHC services. Nevertheless, this study underscores much-needed provider views on utilizing state tobacco quitlines within FQHCs.

## CONCLUSIONS

FQHCs play a crucial role in serving low-income patients who face a disproportionate burden of tobacco use. While barriers to the direct provision of smoking cessation treatments are well-documented, state tobacco quitlines present an opportunity to enhance the availability of such treatments. Improving provider education, strengthening patient motivation, streamlining referral processes, leveraging information exchange systems, and using patient portals to monitor and facilitate engagement are essential to increase referrals to quitlines. By addressing these areas, FQHCs can support ongoing efforts to reduce tobacco use and improve patients’ overall health.

## Data Availability

The data supporting this research are available from the authors on reasonable request.
